# Radiography in Pyorrhœa Alveolaris

**Published:** 1897-02

**Authors:** William Rollins

**Affiliations:** Boston


### Domestic Correspondence.

### RADIOGRAPHY IN PYORRHŒA ALVEOLARIS.

To the Editor:

Sir,—I have been much interested in Dr. W. H. Trueman's article, in the December number, on "Pyorrhœa Alveolaris," one sentence of which I quote: "I am strongly impressed after all that pyorrhœa alveolaris is merely a germ-disease."

I have made a number of radiographs of the alveoli in patients with slight pyorrhœa alveolaris, and find the edges do not extend as far as normal. This, together with the fact that all observers do not consider absorption of bone due to germs, causes me to believe the germ theory not entirely adequate.

As the disease may not necessarily begin at the surface, and as absorption of the alveoli may in some cases be the first symptom, my object in writing this note is to focus attention on this point in order that my observations may be followed up.

William Rollins.

Boston, December 18, 1896.

